# Primary Cell Wall Modifying Proteins Regulate Wall Mechanics to Steer Plant Morphogenesis

**DOI:** 10.3389/fpls.2021.751372

**Published:** 2021-11-17

**Authors:** Dengying Qiu, Shouling Xu, Yi Wang, Ming Zhou, Lilan Hong

**Affiliations:** ^1^Key Laboratory of Nuclear Agricultural Sciences of Ministry of Agriculture and Zhejiang Province, Institute of Nuclear Agricultural Sciences, Zhejiang University, Hangzhou, China; ^2^State Key Laboratory of Plant Physiology and Biochemistry, College of Life Sciences, Zhejiang University, Hangzhou, China

**Keywords:** cell wall remodeling, cell wall modifying proteins, cell wall mechanical properties, plant morphogenesis, pectin methylesterases

## Abstract

Plant morphogenesis involves multiple biochemical and physical processes inside the cell wall. With the continuous progress in biomechanics field, extensive studies have elucidated that mechanical forces may be the most direct physical signals that control the morphology of cells and organs. The extensibility of the cell wall is the main restrictive parameter of cell expansion. The control of cell wall mechanical properties largely determines plant cell morphogenesis. Here, we summarize how cell wall modifying proteins modulate the mechanical properties of cell walls and consequently influence plant morphogenesis.

## Introduction

Plant development is not only principally orchestrated by networks of biochemical signals, but also affected by biophysical restraints from internal cells and external environmental signals. Emerging evidence manifests that mechanical forces act as instructive signals to control plant morphogenesis (Sampathkumar et al., 2014; Bidhendi and Geitmann, 2019). It is widely assumed that plant cell expansion results from irreversible yielding (see Table 1 for a glossary of the biomechanical terms used in this review) of the cell walls to high internal turgor pressure (Cosgrove, 1993; Boudon et al., 2015). While turgor pressure is the driving force behind cell growth, the parameter solely responsible for the control of cell expansion is the extensibility of the cell wall (Baskin, 2005; Geitmann and Ortega, 2009; Boudaoud, 2010).

**Table 1:** Glossary of the terms for cell wall mechanics often used in the context of plant morphogenesis.

Cell wall mechanical properties	Physical properties of the cell wall that determine its behavior upon exposure to deforming forces resulting from pressure, tension, or compression	
Yielding	Material deforming when the applied force exceeds well-defined threshold	
Turgor pressure	A hydrostatic pressure generated by the water pushing the plasma membrane and plant cell wall	
Wall extensibility	Property of the cell wall to be deformed irreversibly under a deforming load, for example, that caused by turgor	
Stiffening	Enhancing the mechanical strength of the cell wall, resulting from the modification of the biochemical configuration	
Softening	Weakening the mechanical strength of the cell wall, resulting from the modification of the biochemical configuration	
Plasticity	The quality or state of being plastic, especially capacity for being deformed or altered. With plastic deformation, materials do not return to their original shape after the pressure on them being removed	
Cell wall rigidity	The apparent rigidity of the cell wall results from turgor pressure while cell wall polymers and bonds density increase is internal cause	
Loosening	Rearrangement of the cell wall polymers facilitating the load-induced extension of the cell wall material	
Creep	Slow, time-dependent, and irreversible extension of the cell wall	
Wall rheology	The study of the flow and deformation of walls in response to an applied force

The cell wall is crucial for many processes of plant development. Besides being a barrier to protect from and interact with the environment, the cell wall also determines the mechanical strength of plant structures (Vaahtera et al., 2019). The tight spatiotemporal regulation of cell wall mechanics is essential for proper morphogenesis at cellular and tissue levels (Dumais et al., 2006; Boudon et al., 2015; Bidhendi and Geitmann, 2016). Cell walls commonly are classified into two major types: primary walls and secondary walls. The primary wall is produced in growing and in dividing cells and plays a prominent role in growth and development and hence is the focus of this review.

**Figure 1:**
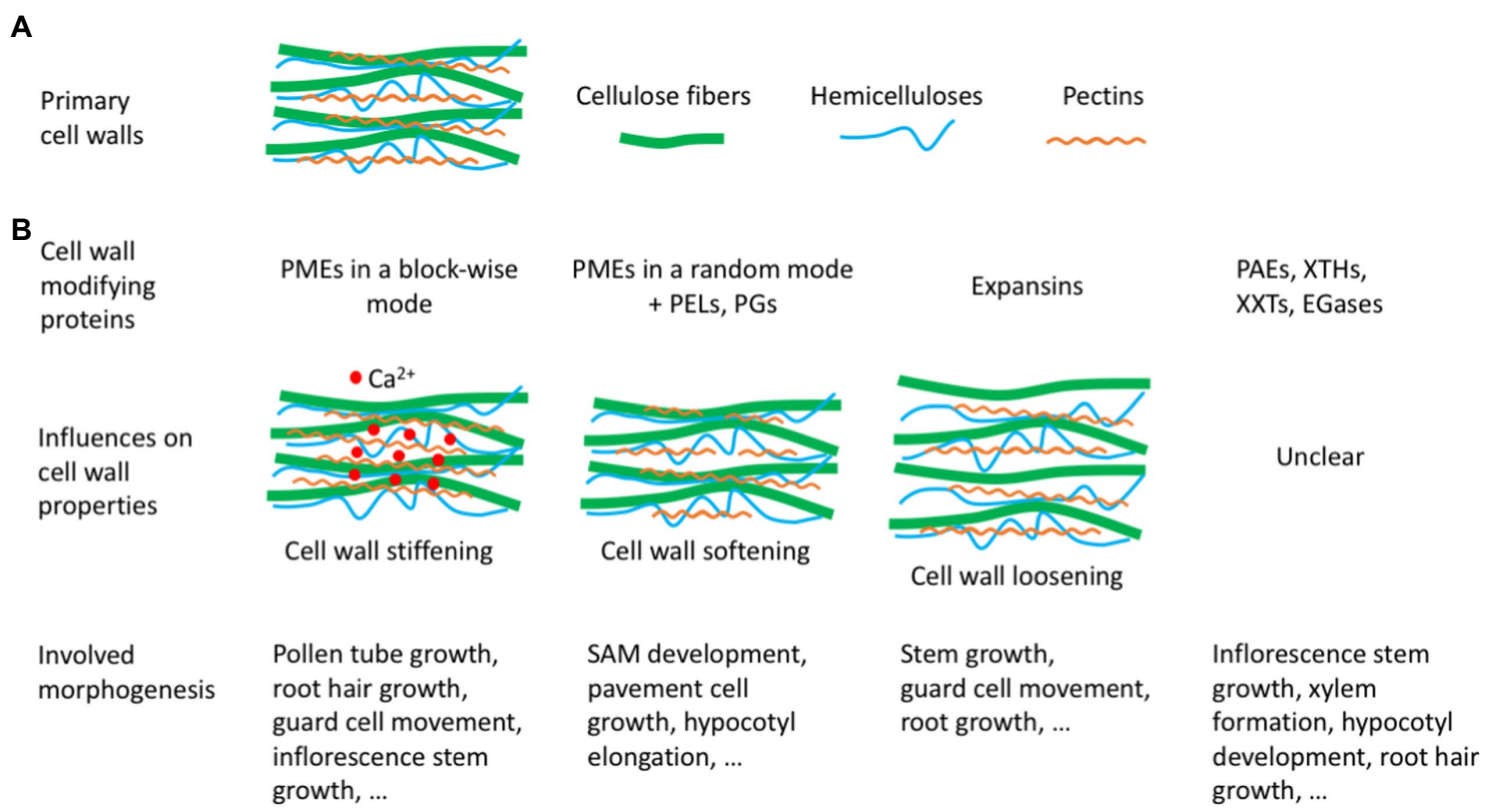
Hypothetical models on the action of cell wall modifying proteins. **(A)** A simplified schematic depicting the structure of the primary cell wall. **(B)** The potential action mechanisms of different cell wall modifying proteins. PMEs, pectin methylesterases; PELs, pectate lyases; PGs, polygalacturonases; PAEs, pectin acetyl esterases, XTHs, xyloglucan endotransglucosylases/hydrolases; XXTs, xylosyltransferases; EGases, endo-1,4-β-glucanases.

Primary cell walls can be described as composite materials made of cellulose microfibrils tethered by hemicelluloses and embedded within pectins and structural proteins (Figure 1A; Cosgrove, 2016; Amos and Mohnen, 2019; Chebli et al., 2021). Cellulose microfibrils are considered to be the main load-bearing components and are extremely stable and usually undergo negligible turnover or breakdown during cell wall growth (Cosgrove, 1997). Pectins and hemicelluloses, the other two primary load-bearing components in the plant cell wall, are constantly remodeled to adjust to the morphological changes of cells during plant development. These remodeling processes are tightly regulated by a plethora of agents (e.g., proteins, enzymes, and ions) spatiotemporally (Cosgrove, 2005). In this review, we will discuss how cell wall modifying proteins modulate mechanical properties of primary walls to influence plant morphogenesis.

## Pectin Modifying Enzymes

Pectin-related cell wall modifications increasingly emerge as an important factor influencing plant morphogenesis. Pectins are a diverse class of galacturonic acid-rich polysaccharides. They are the most complex cell wall components and have a major impact on the physical and chemical characteristics of the cell wall, both structurally and functionally (Wolf and Greiner, 2012). The most abundant pectins are homogalacturonans (HGs), which account for greater than 60% of pectins in the plant cell wall (Caffall and Mohnen, 2009). HGs are polymerized in the Golgi apparatus by glycosyl transferases, substituted with methyl groups at the C6 position and secreted to the cell wall in a highly methylesterified state (Zhang and Staehelin, 1992; Cosgrove, 2005; Sterling et al., 2006; Driouich et al., 2012; Wolf and Greiner, 2012). During plant development, pectins undergo extensive modifications with accompanying changes in their physical and chemical properties.

Demethylesterification by pectin methylesterases (PMEs) is the most extensively studied type of pectin modification. In the cell wall, PMEs can remove the methyl groups of HGs at the C6 position and significantly alter the physical properties of the pectin polymer, thereby changing cell wall mechanics (Wolf et al., 2009). In the Arabidopsis inflorescence stem, cell wall polysaccharide composition and dynamics analyses by solid-state nuclear magnetic resonance spectroscopy coupled with growth tracking indicated a correlation between pectin methylesterification levels and organ growth. It was found that the cell wall of the fastest growing part of the inflorescence stem had higher pectin contents and a higher degree of methylesterification, compared with other stem segments (Phyo et al., 2017).

The quantification of pectin methylesterification state has been greatly promoted by an array of pectin-specific antibodies that specifically recognize various pectin methylesterification epitopes (Clausen et al., 2003; Qi et al., 2017; Schoenaers et al., 2018; Jonsson et al., 2021). Meanwhile, cell wall mechanical properties can be directly measured by atomic force microscopy (AFM) and microindentation at high resolution. The mechanical properties of the cell wall measured by AFM or microindentation can be correlated with the pectin methylesterification identified by pectin-specific antibodies in the same tissue. Numerous studies indicate that there is a strong correlation between pectin antibody labeling, deduced mechanical properties, and actual stiffness measurements of cell walls (Bosch and Hepler, 2005; Parre and Geitmann, 2005; Zerzour et al., 2009; Fayant et al., 2010; Qi et al., 2017; Bou Daher et al., 2018; Wang et al., 2020).

Studies in pollen tubes have shown that a low level of pectin methylesterification was often associated with stiffer walls and the cessation of growth (Bosch and Hepler, 2005; Parre and Geitmann, 2005; Sanati Nezhad et al., 2014). During the growth of the pollen tube, the tip is enriched with highly methylesterificated pectins and in the shank pectin methylesterification is reduced (Zerzour et al., 2009). When PME activity was elevated, cell wall stiffening occurred and pollen tube growth was reduced or blocked (Bosch and Hepler, 2005; Parre and Geitmann, 2005; Roeckel et al., 2008). In a similar manner, overexpression of a PME inhibitor (PMEI) resulted in increased pollen tube elongation (Roeckel et al., 2008).

However, research in the shoot apical meristem (SAM) of Arabidopsis displayed different results about the degree of pectin methylesterification and growth rate, contrary to the situation in pollen tubes. SAM sections labeled with specific antibodies for demethylesterified pectins showed that the degree of methylesterification appeared to be relatively high in the meristem dome, yet incipient primordia were strongly labeled, indicating that PME activity is very strong at these sites (Peaucelle et al., 2008). Furthermore, elevated PME levels caused by genetic modifications increased the number of primordia and disrupted the phyllotactic pattern. Consistently, ectopic expression of PMEI arrested the development of new primordia, presumably by hardening the cell wall through inhibiting pectin demethylesterification. Most intriguingly, the deposition of sepharose beads with PME on the wild-type meristem resulted in a new primordium which developed into a normal floral meristem because of the softening of cell walls by PMEs. Meanwhile, the meristem of a primordia marker line treated with PME beads displayed strong disruption in phyllotactic patterning (Peaucelle et al., 2008, 2011; Wolf and Greiner, 2012). In summary, these results evidently suggest that pectin demethylesterification mediated by PMEs is necessary and sufficient for cell wall softening and primordium initiation in Arabidopsis SAMs.

The contradictory effects of PME-mediated pectin demethylesterification on cell wall stiffness and consequently on cell growth in SAMs and pollen tubes could be related to PME’s different action modes (Bidhendi and Geitmann, 2016). PMEs are proposed to have two action modes: the block-wise fashion and the random fashion (Figure 1B). In the block-wise fashion, PME isoforms create long blocks of contiguous free carboxyl groups susceptible to interact with Ca^2+^, thus forming a stiff pectate network (e.g., the egg box configuration with multiple HG chains crosslinking). In the random fashion, PME isoforms operating randomly lead to a random demethylesterification of HGs, accordingly promoting the action of pectin depolymerases, such as pectate lyases (PELs), which results in the degradation of pectins and cell wall softening (Liners et al., 1992; Micheli, 2001; Willats et al., 2001; Pelloux et al., 2007). Experiments on cell-free strips of onion epidermal walls also demonstrated that PME-mediated pectin demethylesterification had opposing effects on cell wall properties. Through measuring both wall biomechanics with surface indentation and wall extensibility with tensile tests, researchers found that PME treatment alone softened cell walls, but would reduce wall plasticity in the presence of abundant Ca^2+^ (Wang et al., 2020).

The action patterns of PMEs depend on many factors, including pH of the cell wall, the initial methylesterification of the pectins, as well as cations, among which Ca^2+^ plays a significant role. Pollen tube growth is remarkably influenced by Ca^2+^ (Sanati Nezhad et al., 2014). Elevating Ca^2+^ concentration interrupted pollen tube growth, whereas removal of Ca^2+^ from the medium caused the burst of pollen tubes (Picton and Steer, 1983; Hepler and Winship, 2010), suggesting that PMEs in the pollen tubes might work in the block-wise fashion. In other cells whose physiological function and growth are also under the influence of Ca^2+^ dynamics, such as root hair cells and guard cells, PMEs act in a similar mode. In root hairs, elevated PME activity of the cell walls also led to growth inhibition (Schoenaers et al., 2018). Likewise, in guard cells, the regions with high stiffness accumulated more demethylesterified homogalacturonic polymers and the increase of methylesterified pectins resulted in a smaller dynamic range of guard cell movement (Amsbury et al., 2016; Carter et al., 2017). In the meanwhile, in tissues with limited Ca^2+^ distribution, such as hypocotyls, studies echoed the results obtained in the SAM (Pelletier et al., 2010; Bou Daher et al., 2018). Experiments with isolated onion epidermal walls also manifested that abundant Ca^2+^ could negate PME-mediated wall softening and lead to reduced wall plasticity (Wang et al., 2020).

In a word, PME action is two-sided and has potential to either enhance or lower cell wall rigidity. Although cell wall softening caused by PMEs alone does not lead to cell wall loosening (Zhang et al., 2019; Wang et al., 2020), the cooperation of PMEs with other cell wall digesting enzymes may lead to more profound changes in pectin conformation and cell wall properties.

Meanwhile, cutting-edge imaging techniques advance our understandings on pectin conformation. A recent study reported nanofibrillar pectin structures (HG nanofilaments) observed with super-resolution microscopy and cryo-scanning electron microscopy in Arabidopsis pavement cells. The authors found that demethylation by PMEs led to local radial swelling of the HG nanofilaments and tissue expansion. By means of computational modeling, they proposed that the demethylation of HGs could cause local wall expansion in the absence of turgor-derived force (Haas et al., 2020). This finding challenged current models that cell expansion is driven by turgor pressure acting on the cell wall, although great controversies and discussions had been aroused by the results and interpretations of this work (Cosgrove and Anderson, 2020).

Besides methylesterification, acetylation is another important modification of pectins. Pectin acetylation has been shown to play significant roles in Arabidopsis development. The Reduced Wall Acetylation (RWA) protein family is involved in the cell wall acetylation. The single mutant, *rwa2*, had an about 20% decrease, and the quadruple mutant, *rwa1 rwa2 rwa3 rwa4*, showed a 63% reduction in cell wall O-acetylation compared with the wild type, respectively, and they were associated with severe dwarfism (Manabe et al., 2011, 2013). Mutant lines of pectin acetyl esterase (PAE) encoding genes *PAE8* and *PAE9* also displayed reduced inflorescence stem growth (de Souza et al., 2014).

In addition to the pectin modification enzymes, the structure and composition of pectins are modulated by pectin depolymerases, including PELs and polygalacturonases (PGs). PELs, which degrade demethylesterified pectin, play crucial roles in leaf growth and senescence in rice (Wu et al., 2013; Leng et al., 2017), pollen wall development in Chinese cabbage (Jiang et al., 2014a,b; Rhee et al., 2015), and fruit ripening and softening in tomato (Uluisik et al., 2016; Yang et al., 2017; Wang et al., 2018, 2019; Uluisik and Seymour, 2020). In rice, the mutation of a PEL precursor gene *DWARF AND EARLY SENESCENCE LEAF1* (*DEL1*) led to dwarfism and an early senescence leaf phenotype. *DEL1* mutation decreased the total PEL enzymatic activity, increased the degree of methylesterified HGs, and altered the cell wall composition and structure in culms, suggesting a role of PELs in the maintenance of cell division and the induction of leaf senescence (Leng et al., 2017).

PGs catalyze the hydrolysis and disassembly of pectin and have potential roles in plant development. Transgenic strawberries overexpressing an antisense sequence of a strawberry PG gene *FaPG1* produced fruits significantly firmer than wild type, indicating that PGs play a key role on fruit softening (Garcia-Gago et al., 2009; Quesada et al., 2009; Pose et al., 2013). PGs are also involved in the development of Arabidopsis seed coat. Overexpressing *ARABIDOPSIS DEHISCENCE ZONE POLYGALACTURONASE2* (*ADPG2*) in Arabidopsis seed coat epidermal cells resulted in abnormal cell morphogenesis with disrupted cell-cell adhesion and signs of early cell death (McGee et al., 2021).

In summary, a strict regulation of the complex process of pectin modification is required for creating appropriate cell wall mechanical properties to maintain proper cell and organ shape. This regulation relies on multiple factors, such as the types of pectin modification enzymes, the local concentration of Ca^2+^, and the presence of pectin digesting enzymes and enzyme inhibitors (such as PMEIs; Palin and Geitmann, 2012; Atmodjo et al., 2013; Sanati Nezhad and Geitmann, 2013; Sanati Nezhad et al., 2014; Wang et al., 2020).

## Hemicellulose Modifying Enzymes

Xyloglucans are the main components of hemicelluloses in the primary walls of dicots and non-graminaceous monocots. Plant cells typically expand 10- to 100-fold in volume before reaching maturity. In extreme cases, cells may enlarge more than 10,000-fold in volume (e.g., xylem vessel elements). The cell wall typically undergoes great expansion without losing its mechanical integrity or becoming thinner. Thus, newly synthesized polymers are integrated into the wall without destabilizing it (Chebli and Geitmann, 2017; Chebli et al., 2021). Although exactly how this integration is accomplished is unclear, it was proposed that self-assembly (Cannon et al., 2008) and enzyme-mediated wall assembly (Holland et al., 2020; Stratilova et al., 2020) play important roles in the integration process of cell walls. With regard to the self-assembly of cell walls, readers could find details in this review (Murugesan et al., 2015). Prime candidates carrying out the enzyme-mediated wall assembly are xyloglucan endotransglucosylases/hydrolases (XTHs).

XTH proteins have two enzymologically different activities: xyloglucan endotransglucosylase (XET) activity and xyloglucan endohydrolase (XEH) activity. The XET activity plays a major role in the enzyme-mediated wall assembly. Such activity has the ability to cut the backbone of a xyloglucan and to join one end of the cut xyloglucan with the end of an acceptor xyloglucan, thereby integrating newly synthesized xyloglucans into the wall (Nishitani, 1997; Thompson and Fry, 2001). It has been postulated that XTHs carry out various functions, including maintaining meristem geometry and phyllotaxis, enhancing freezing tolerance, regulating xylem formation and hypocotyl growth (Bourquin et al., 2002; Matsui et al., 2005; Takahashi et al., 2021). For instance, the *xth27* mutant had a developmental phenotype in xylem development, with fewer tertiary veins (Matsui et al., 2005). And the *xth19* mutant showed reduced freezing tolerance, which was associated with alterations in cell wall composition and structure (Takahashi et al., 2021).

Although previous research found that xyloglucan deficiency had only subtle effects on growth, recent investigations on enzymes involved in xyloglucan biosynthesis, such as xylosyltransferases (XXT), manifested that xyloglucans were required for plant development (Cavalier et al., 2008; Park and Cosgrove, 2012; Xiao et al., 2016; Zhao et al., 2019). The Arabidopsis *XXT* gene double mutant *xxt1 xxt2* is smaller than the wild type but otherwise nearly normal during its development. However, more sophisticated biochemical assays demonstrated that the mutant had more compliant and extensible cell walls than the wild type and developed short root hairs with bulging bases (Cavalier et al., 2008; Park and Cosgrove, 2012; Aryal et al., 2020). In addition, a recent study found that *xxt1 xxt2* mutants exhibited significant defects in hypocotyl hook development, with a smaller hook angle and hooks opening earlier than the wild type (Aryal et al., 2020), supporting the role of xyloglucans in strengthening primary walls.

## Other Cell Wall Modifying Proteins

In addition to the enzymes discussed above, other proteins, such as endo-1,4-β-glucanases and expansins, also play crucial roles in modifying the cell wall and exerting influences on plant morphogenesis. Plant endo-β-1,4-glucanases hydrolyze β-1, 4-glucan bonds, such as those found in cellulose and xyloglucans. They are involved in xyloglucan hydrolysis during fruit ripening and abscission (Hayashi, 1989; Kemmerer and Tucker, 1994), and cellulose synthesis (Tsabary et al., 2003; Glass et al., 2015). Expansins are proteins that can mediate cell wall loosening and are considered key regulators of extension growth. The molecular basis for expansin action on wall rheology is still unclear, but most evidence indicates that expansins cause wall expansion by loosening non-covalent adhesion between wall polysaccharides (Cosgrove, 2000; Li and Cosgrove, 2001). The molecular structures, potential working mechanisms, and functions of expansins in plant development have been elaborated (Cosgrove, 2015; Marowa et al., 2016), so they will not be discussed in detail here.

## Conclusion and Perspectives

The growth, development, and survival of a plant depend on the flexibility and integrity of its cell walls. Cell walls are under constant remodeling in response to developmental cues and environmental challenges. Modifications and regulations of cell wall polymers, especially pectins and hemicelluloses, by various modifying proteins play a tremendous role in cell wall remodeling. Additionally, alternations in the content and composition of wall polymers, as well as interactions between wall polymers and wall structural proteins, also contribute to cell wall remodeling. How these multi-layers of regulations are coordinated to achieve appropriate cell wall structure and properties is still not completely understood. Burgeoning techniques, such as super-resolution microscopy, computational simulation, and polysaccharide profiling, might facilitate further deciphering of the mechanism by which cells integrate all the signals and regulations concerning cell wall remodeling.

## Author Contributions

DQ, SX, YW, and LH wrote the manuscript. LH and MZ edited the manuscript. All authors contributed to the article and approved the submitted version.

## Funding

This work was supported by the National Natural Science Foundation of China grant no. 32070853 (LH), Hundred-Talent Program of Zhejiang University (LH and MZ) and the Fundamental Research Funds for the Central Universities grant no. 2020QNA6006 (MZ).

## Conflict of Interest

The authors declare that the research was conducted in the absence of any commercial or financial relationships that could be construed as a potential conflict of interest.

## Publisher’s Note

All claims expressed in this article are solely those of the authors and do not necessarily represent those of their affiliated organizations, or those of the publisher, the editors and the reviewers. Any product that may be evaluated in this article, or claim that may be made by its manufacturer, is not guaranteed or endorsed by the publisher.

## References

[ref1] Amos R. A. Mohnen D. (2019). Critical review of plant Cell Wall matrix polysaccharide Glycosyltransferase activities verified by heterologous protein expression. Front. Plant Sci. 10:10. doi: 10.3389/fpls.2019.00915 31379900 PMC6646851

[ref2] Amsbury S. Hunt L. Elhaddad N. Baillie A. Lundgren M. Verhertbruggen Y.. (2016). Stomatal function requires pectin de-methyl-esterification of the guard cell wall. Curr. Biol. 26, 2899–2906. doi: 10.1016/j.cub.2016.08.021, PMID: 27720618 PMC5106435

[ref3] Aryal B. Jonsson K. Baral A. Sancho-Andres G. Routier-Kierzkowska A. L. Kierzkowski D.. (2020). Interplay between cell wall and auxin mediates the control of differential cell elongation during apical hook development. Curr. Biol. 30, 1733–1739.e3. doi: 10.1016/j.cub.2020.02.055, PMID: 32197084

[ref4] Atmodjo M. A. Hao Z. Mohnen D. (2013). Evolving views of pectin biosynthesis. Annu. Rev. Plant Biol. 64, 747–779. doi: 10.1146/annurev-arplant-042811-105534 23451775

[ref5] Baskin T. I. (2005). Anisotropic expansion of the plant cell wall. Annu. Rev. Cell Dev. Biol. 21, 203–222. doi: 10.1146/annurev.cellbio.20.082503.103053 16212493

[ref6] Bidhendi A. J. Geitmann A. (2016). Relating the mechanics of the primary plant cell wall to morphogenesis. J. Exp. Bot. 67, 449–461. doi: 10.1093/jxb/erv535, PMID: 26689854

[ref7] Bidhendi A. J. Geitmann A. (2019). Geometrical details matter for mechanical modeling of cell morphogenesis. Dev. Cell 50, 117–125. doi: 10.1016/j.devcel.2019.05.001, PMID: 31265810

[ref8] Bosch M. Hepler P. K. (2005). Pectin methylesterases and pectin dynamics in pollen tubes. Plant Cell 17, 3219–3226. doi: 10.1105/tpc.105.037473, PMID: 16322606 PMC1315365

[ref9] Bou Daher F. Chen Y. Bozorg B. Clough J. Jonsson H. Braybrook S. A. (2018). Anisotropic growth is achieved through the additive mechanical effect of material anisotropy and elastic asymmetry. Elife 7:e38161. doi: 10.7554/eLife.38161 30226465 PMC6143341

[ref10] Boudaoud A. (2010). An introduction to the mechanics of morphogenesis for plant biologists. Trends Plant Sci. 15, 353–360. doi: 10.1016/j.tplants.2010.04.002, PMID: 20427223

[ref11] Boudon F. Chopard J. Ali O. Gilles B. Hamant O. Boudaoud A.. (2015). A computational framework for 3D mechanical modeling of plant morphogenesis with cellular resolution. PLoS Comput. Biol. 11:e1003950. doi: 10.1371/journal.pcbi.1003950, PMID: 25569615 PMC4288716

[ref12] Bourquin V. Nishikubo N. Abe H. Brumer H. Denman S. Eklund M.. (2002). Xyloglucan endotransglycosylases have a function during the formation of secondary cell walls of vascular tissues. Plant Cell 14, 3073–3088. doi: 10.1105/tpc.007773, PMID: 12468728 PMC151203

[ref13] Caffall K. H. Mohnen D. (2009). The structure, function, and biosynthesis of plant cell wall pectic polysaccharides. Carbohydr. Res. 344, 1879–1900. doi: 10.1016/j.carres.2009.05.021, PMID: 19616198

[ref14] Cannon M. C. Terneus K. Hall Q. Tan L. Wang Y. Wegenhart B. L.. (2008). Self-assembly of the plant cell wall requires an extensin scaffold. Proc. Natl. Acad. Sci. U. S. A. 105, 2226–2231. doi: 10.1073/pnas.0711980105, PMID: 18256186 PMC2538902

[ref15] Carter R. Woolfenden H. Baillie A. Amsbury S. Carroll S. Healicon E.. (2017). Stomatal opening involves polar, not radial, stiffening of guard cells. Curr. Biol. 27, 2974–2983. doi: 10.1016/j.cub.2017.08.006, PMID: 28943087 PMC5640513

[ref16] Cavalier D. M. Lerouxel O. Neumetzler L. Yamauchi K. Reinecke A. Freshour G.. (2008). Disrupting two Arabidopsis thaliana xylosyltransferase genes results in plants deficient in xyloglucan, a major primary cell wall component. Plant Cell 20, 1519–1537. doi: 10.1105/tpc.108.059873, PMID: 18544630 PMC2483363

[ref17] Chebli Y. Bidhendi A. J. Kapoor K. Geitmann A. (2021). Cytoskeletal regulation of primary plant cell wall assembly. Curr. Biol. 31, R681–R695. doi: 10.1016/j.cub.2021.03.092, PMID: 34033798

[ref18] Chebli Y. Geitmann A. (2017). Cellular growth in plants requires regulation of cell wall biochemistry. Curr. Opin. Cell Biol. 44, 28–35. doi: 10.1016/j.ceb.2017.01.002, PMID: 28131101

[ref19] Clausen M. H. Willats W. G. T. Knox J. P. (2003). Synthetic methyl hexagalacturonate hapten inhibitors of antihomogalacturonan monoclonal antibodies LM7, JIM5 and JIM7. Carbohydr. Res. 338, 1797–1800. doi: 10.1016/s0008-6215(03)00272-6, PMID: 12892947

[ref20] Cosgrove D. J. (1993). Wall extensibility: its nature, measurement and relationship to plant cell growth. New Phytol. 124, 1–23. doi: 10.1111/j.1469-8137.1993.tb03795.x 11537718

[ref21] Cosgrove D. J. (1997). Relaxation in a high-stress environment: The molecular bases of extensible cell walls and cell enlargement. Plant Cell 9, 1031–1041. doi: 10.1105/tpc.9.7.1031, PMID: 9254929 PMC156977

[ref22] Cosgrove D. J. (2000). Loosening of plant cell walls by expansins. Nature 407, 321–326. doi: 10.1038/35030000, PMID: 11014181

[ref23] Cosgrove D. J. (2005). Growth of the plant cell wall. Nat. Rev. Mol. Cell Biol. 6, 850–861. doi: 10.1038/nrm1746, PMID: 16261190

[ref24] Cosgrove D. J. (2015). Plant expansins: diversity and interactions with plant cell walls. Curr. Opin. Plant Biol. 25, 162–172. doi: 10.1016/j.pbi.2015.05.014, PMID: 26057089 PMC4532548

[ref25] Cosgrove D. J. (2016). Plant cell wall extensibility: connecting plant cell growth with cell wall structure, mechanics, and the action of wall-modifying enzymes. J. Exp. Bot. 67, 463–476. doi: 10.1093/jxb/erv511, PMID: 26608646

[ref26] Cosgrove D. J. Anderson C. T. (2020). Plant cell growth: do pectins drive lobe formation in Arabidopsis pavement cells? Curr. Biol. 30, R660–R662. doi: 10.1016/j.cub.2020.04.007, PMID: 32516619

[ref27] de Souza A. Hull P. A. Gille S. Pauly M. (2014). Identification and functional characterization of the distinct plant pectin esterases PAE8 and PAE9 and their deletion mutants. Planta 240, 1123–1138. doi: 10.1007/s00425-014-2139-6, PMID: 25115560 PMC4200376

[ref28] Driouich A. Follet-Gueye M. L. Bernard S. Kousar S. Chevalier L. Vicre-Gibouin M.. (2012). Golgi-mediated synthesis and secretion of matrix polysaccharides of the primary Cell Wall of higher plants. Front. Plant Sci. 3:79. doi: 10.3389/fpls.2012.00079 22639665 PMC3355623

[ref29] Dumais J. Shaw S. L. Steele C. R. Long S. R. Ray P. M. (2006). An anisotropic-viscoplastic model of plant cell morphogenesis by tip growth. Int. J. Dev. Biol. 50, 209–222. doi: 10.1387/ijdb.052066jd, PMID: 16479489

[ref30] Fayant P. Girlanda O. Chebli Y. Aubin C. E. Villemure I. Geitmann A. (2010). Finite element model of polar growth in pollen tubes. Plant Cell 22, 2579–2593. doi: 10.1105/tpc.110.075754, PMID: 20699395 PMC2947179

[ref31] Garcia-Gago J. A. Pose S. Munoz-Blanco J. Quesada M. A. Mercado J. A. (2009). The polygalacturonase FaPG1 gene plays a key role in strawberry fruit softening. Plant Signal. Behav. 4, 766–768. doi: 10.4161/psb.4.8.9167, PMID: 19820312 PMC2801395

[ref32] Geitmann A. Ortega J. K. E. (2009). Mechanics and modeling of plant cell growth. Trends Plant Sci. 14, 467–478. doi: 10.1016/j.tplants.2009.07.006, PMID: 19717328

[ref33] Glass M. Barkwill S. Unda F. Mansfield S. D. (2015). Endo-beta-1,4-glucanases impact plant cell wall development by influencing cellulose crystallization. J. Integr. Plant Biol. 57, 396–410. doi: 10.1111/jipb.12353, PMID: 25756224

[ref34] Haas K. T. Wightman R. Meyerowitz E. M. Peaucelle A. (2020). Pectin homogalacturonan nanofilament expansion drives morphogenesis in plant epidermal cells. Science 367, 1003–1007. doi: 10.1126/science.aaz5103, PMID: 32108107 PMC7932746

[ref35] Hayashi T. (1989). Xyloglucans in the primary-cell wall. Annu. Rev. Plant Physiol. Plant Mol. Biol. 40, 139–168. doi: 10.1146/annurev.pp.40.060189.001035

[ref36] Hepler P. K. Winship L. J. (2010). Calcium at the cell wall-cytoplast interface. J. Integr. Plant Biol. 52, 147–160. doi: 10.1111/j.1744-7909.2010.00923.x, PMID: 20377677

[ref37] Holland C. Simmons T. J. Meulewaeter F. Hudson A. Fry S. C. (2020). Three highly acidic equisetum XTHs differ from hetero-trans-beta-glucanase in donor substrate specificity and are predominantly xyloglucan homo-transglucosylases. J. Plant Physiol. 251:e153210. doi: 10.1016/j.jplph.2020.153210, PMID: 32544741

[ref38] Jiang J. Yao L. Yu Y. Liang Y. Jiang J. Ye N.. (2014a). PECTATE LYASE-LIKE 9 from Brassica campestris is associated with intine formation. Plant Sci. 229, 66–75. doi: 10.1016/j.plantsci.2014.08.008, PMID: 25443834

[ref39] Jiang J. Yao L. Yu Y. Lv M. Miao Y. Cao J. (2014b). PECTATE LYASE-LIKE10 is associated with pollen wall development in Brassica campestris. J. Integr. Plant Biol. 56, 1095–1105. doi: 10.1111/jipb.12209, PMID: 24773757

[ref40] Jonsson K. Lathe R. S. Kierzkowski D. Routier-Kierzkowska A. L. Hamant O. Bhalerao R. P. (2021). Mechanochemical feedback mediates tissue bending required for seedling emergence. Curr. Biol. 31, 1154–1164. doi: 10.1016/j.cub.2020.12.016, PMID: 33417884

[ref41] Kemmerer E. C. Tucker M. L. (1994). Comparative-study of cellulases associated with adventitious root initiation, apical buds, and leaf, flower, and pod abscission zones in soybean. Plant Physiol. 104, 557–562. doi: 10.1104/pp.104.2.557, PMID: 8159787 PMC159231

[ref42] Leng Y. Yang Y. Ren D. Huang L. Dai L. Wang Y.. (2017). A rice PECTATE LYASE-LIKE gene is required for plant growth and leaf senescence. Plant Physiol. 174, 1151–1166. doi: 10.1104/pp.16.01625, PMID: 28455404 PMC5462006

[ref43] Li L. C. Cosgrove D. J. (2001). Grass group I pollen allergens (beta-expansins) lack proteinase activity and do not cause wall loosening via proteolysis. Eur. J. Biochem. 268, 4217–4226. doi: 10.1046/j.1432-1327.2001.02336.x, PMID: 11488915

[ref44] Liners F. Thibault J. F. Vancutsem P. (1992). Influence of the degree of polymerization of oligogalacturonates and of esterification pattern of pectin on their recognition by monoclonal-antibodies. Plant Physiol. 99, 1099–1104. doi: 10.1104/pp.99.3.1099, PMID: 16668976 PMC1080589

[ref45] Manabe Y. Nafisi M. Verhertbruggen Y. Orfila C. Gille S. Rautengarten C.. (2011). Loss-of-function mutation of REDUCED WALL ACETYLATION2 in Arabidopsis leads to reduced cell wall ACETYLATION and increased resistance to botrytis cinerea. Plant Physiol. 155, 1068–1078. doi: 10.1104/pp.110.168989, PMID: 21212300 PMC3046569

[ref46] Manabe Y. Verhertbruggen Y. Gille S. Harholt J. Chong S. L. Pawar P. M. A.. (2013). Reduced Wall acetylation proteins play vital and distinct roles in cell wall O-acetylation in Arabidopsis. Plant Physiol. 163, 1107–1117. doi: 10.1104/pp.113.225193, PMID: 24019426 PMC3813637

[ref47] Marowa P. Ding A. Kong Y. (2016). Expansins: roles in plant growth and potential applications in crop improvement. Plant Cell Rep. 35, 949–965. doi: 10.1007/s00299-016-1948-4, PMID: 26888755 PMC4833835

[ref48] Matsui A. Yokoyama R. Seki M. Ito T. Shinozaki K. Takahashi T.. (2005). AtXTH27 plays an essential role in cell wall modification during the development of tracheary elements. Plant J. 42, 525–534. doi: 10.1111/j.1365-313X.2005.02395.x, PMID: 15860011

[ref49] McGee R. Dean G. H. Wu D. Zhang Y. Mansfield S. D. Haughn G. W. (2021). Pectin modification in seed coat mucilage by in vivo expression of rhamnogalacturonan-I- and homogalacturonan-degrading enzymes. Plant Cell Physiol. 1:pcab077. doi: 10.1093/pcp/pcab077 34059917

[ref50] Micheli F. (2001). Pectin methylesterases: cell wall enzymes with important roles in plant physiology. Trends Plant Sci. 6, 414–419. doi: 10.1016/s1360-1385(01)02045-3, PMID: 11544130

[ref51] Murugesan Y. K. Pasini D. Rey A. D. (2015). Self-assembly mechanisms in plant cell wall components. J. Renewable Mat. 3, 56–72. doi: 10.7569/jrm.2014.634124

[ref52] Nishitani K. (1997). The role of endoxyloglucan transferase in the organization of plant cell walls. Int. Rev. Cytol. 173, 157–206. doi: 10.1016/s0074-7696(08)62477-8 9127953

[ref53] Palin R. Geitmann A. (2012). The role of pectin in plant morphogenesis. Biosystems 109, 397–402. doi: 10.1016/j.biosystems.2012.04.006, PMID: 22554809

[ref54] Park Y. B. Cosgrove D. J. (2012). Changes in cell wall biomechanical properties in the xyloglucan-deficient xxt1/xxt2 mutant of Arabidopsis. Plant Physiol. 158, 465–475. doi: 10.1104/pp.111.189779, PMID: 22108526 PMC3252101

[ref55] Parre E. Geitmann A. (2005). Pectin and the role of the physical properties of the cell wall in pollen tube growth of Solanum chacoense. Planta 220, 582–592. doi: 10.1007/s00425-004-1368-5, PMID: 15449057

[ref56] Peaucelle A. Braybrook S. A. Le Guillou L. Bron E. Kuhlemeier C. Hoefte H. (2011). Pectin-induced changes in cell wall mechanics underlie organ initiation in Arabidopsis. Curr. Biol. 21, 1720–1726. doi: 10.1016/j.cub.2011.08.057, PMID: 21982593

[ref57] Peaucelle A. Louvet R. Johansen J. N. Hoefte H. Laufs P. Pelloux J.. (2008). Arabidopsis phyllotaxis is controlled by the methyl-esterification status of cell-wall pectins. Curr. Biol. 18, 1943–1948. doi: 10.1016/j.cub.2008.10.065, PMID: 19097903

[ref58] Pelletier S. Van Orden J. Wolf S. Vissenberg K. Delacourt J. Ndong Y. A.. (2010). A role for pectin de-methylesterification in a developmentally regulated growth acceleration in dark-grown Arabidopsis hypocotyls. New Phytol. 188, 726–739. doi: 10.1111/j.1469-8137.2010.03409.x 20819179

[ref59] Pelloux J. Rusterucci C. Mellerowicz E. J. (2007). New insights into pectin methylesterase structure and function. Trends Plant Sci. 12, 267–277. doi: 10.1016/j.tplants.2007.04.001, PMID: 17499007

[ref60] Phyo P. Wang T. Kiemle S. N. O'Neill H. Pingali S. V. Hong M.. (2017). Gradients in wall mechanics and polysaccharides along growing inflorescence stems. Plant Physiol. 175, 1593–1607. doi: 10.1104/pp.17.01270, PMID: 29084904 PMC5717741

[ref61] Picton J. M. Steer M. W. (1983). Evidence for the role of Ca^2+^ ions in tip extension in pollen tubes. Protoplasma 115, 11–17. doi: 10.1007/bf01293575

[ref62] Pose S. Paniagua C. Cifuentes M. Blanco-Portales R. Quesada M. A. Mercado J. A. (2013). Insights into the effects of polygalacturonase FaPG1 gene silencing on pectin matrix disassembly, enhanced tissue integrity, and firmness in ripe strawberry fruits. J. Exp. Bot. 64, 3803–3815. doi: 10.1093/jxb/ert210, PMID: 23873994 PMC3745733

[ref63] Qi J. Wu B. Feng S. Lu S. Guan C. Zhang X.. (2017). Mechanical regulation of organ asymmetry in leaves. Nat. Plants 3, 724–733. doi: 10.1038/s41477-017-0008-6, PMID: 29150691

[ref64] Quesada M. A. Blanco-Portales R. Pose S. Garcia-Gago J. A. Jimenez-Bermudez S. Munoz-Serrano A.. (2009). Antisense down-regulation of the FaPG1 gene reveals an unexpected central role for polygalacturonase in strawberry fruit softening. Plant Physiol. 150, 1022–1032. doi: 10.1104/pp.109.138297, PMID: 19395408 PMC2689968

[ref65] Rhee S. J. Seo M. Jang Y. J. Cho S. Lee G. P. (2015). Transcriptome profiling of differentially expressed genes in floral buds and flowers of male sterile and fertile lines in watermelon. Bmc Genomics 16:914. doi: 10.1186/s12864-015-2186-9, PMID: 26552448 PMC4640349

[ref66] Roeckel N. Wolf S. Kost B. Rausch T. Greiner S. (2008). Elaborate spatial patterning of cell-wall PME and PMEI at the pollen tube tip involves PMEI endocytosis, and reflects the distribution of esterified and de-esterified pectins. Plant J. 53, 133–143. doi: 10.1111/j.1365-313X.2007.03325.x 17971035

[ref67] Sampathkumar A. Krupinski P. Wightman R. Milani P. Berquand A. Boudaoud A.. (2014). Subcellular and supracellular mechanical stress prescribes cytoskeleton behavior in Arabidopsis cotyledon pavement cells. elife 3:e01967. doi: 10.7554/eLife.01967 24740969 PMC3985187

[ref68] Sanati Nezhad A. Geitmann A. (2013). The cellular mechanics of an invasive lifestyle. J. Exp. Bot. 64, 4709–4728. doi: 10.1093/jxb/ert254, PMID: 24014865

[ref69] Sanati Nezhad A. Packirisamy M. Geitmann A. (2014). Dynamic, high precision targeting of growth modulating agents is able to trigger pollen tube growth reorientation. Plant J. 80, 185–195. doi: 10.1111/tpj.12613, PMID: 25041411

[ref70] Schoenaers S. Balcerowicz D. Breen G. Hill K. Zdanio M. Mouille G.. (2018). The Auxin-regulated CrRLK1L kinase ERULUS controls cell wall composition during root hair tip growth. Curr. Biol. 28, 722–732. doi: 10.1016/j.cub.2018.01.050, PMID: 29478854

[ref71] Sterling J. D. Atmodjo M. A. Inwood S. E. Kolli V. S. K. Quigley H. F. Hahn M. G.. (2006). Functional identification of an Arabidopsis pectin biosynthetic homogalacturonan galacturonosyltransferase. Proc. Natl. Acad. Sci. U. S. A. 103, 5236–5241. doi: 10.1073/pnas.0600120103, PMID: 16540543 PMC1458824

[ref72] Stratilova B. Sestak S. Mravec J. Garajova S. Pakanova Z. Vadinova K.. (2020). Another building block in the plant cell wall: barley xyloglucan xyloglucosyl transferases link covalently xyloglucan and anionic oligosaccharides derived from pectin. Plant J. 104, 752–767. doi: 10.1111/tpj.14964, PMID: 32799357

[ref73] Takahashi D. Johnson K. Hao P. Tuong T. Erban A. Sampathkumar A.. (2021). Cell wall modification by the xyloglucan endotransglucosylase/hydrolase XTH19 influences freezing tolerance after cold and sub-zero acclimation. Plant Cell Environ. 44, 915–930. doi: 10.1111/pce.13953 33190295

[ref74] Thompson J. E. Fry S. C. (2001). Restructuring of wall-bound xyloglucan by transglycosylation in living plant cells. Plant J. 26, 23–34. doi: 10.1046/j.1365-313x.2001.01005.x, PMID: 11359607

[ref75] Tsabary G. Shani Z. Roiz L. Levy I. Riov J. Shoseyov O. (2003). Abnormal 'wrinkled' cell walls and retarded development of transgenic Arabidopsis thaliana plants expressing endo-1,4-beta-glucanase (cel1) antisense. Plant Mol. Biol. 51, 213–224. doi: 10.1023/a:1021162321527, PMID: 12602880

[ref76] Uluisik S. Chapman N. H. Smith R. Poole M. Adams G. Gillis R. B.. (2016). Genetic improvement of tomato by targeted control of fruit softening. Nat. Biotechnol. 34:1072. doi: 10.1038/nbt1016-1072d, PMID: 27727231

[ref77] Uluisik S. Seymour G. B. (2020). Pectate lyases: their role in plants and importance in fruit ripening. Food Chem. 309:125559. doi: 10.1016/j.foodchem.2019.125559, PMID: 31679850

[ref78] Vaahtera L. Schulz J. Hamann T. (2019). Cell wall integrity maintenance during plant development and interaction with the environment. Nat. Plants 5, 924–932. doi: 10.1038/s41477-019-0502-0, PMID: 31506641

[ref79] Wang D. Samsulrizal N. H. Yang C. Allcock N. S. Craigon J. Blanco-Ulate B.. (2019). Characterization of CRISPR mutants targeting genes modulating pectin degradation in ripening tomato. Plant Physiol. 179, 554–557. doi: 10.1104/pp.18.01187 PMC6426429 30459263

[ref80] Wang X. Wilson L. Cosgrove D. J. (2020). Pectin methylesterase selectively softens the onion epidermal wall yet reduces acid-induced creep. J. Exp. Bot. 71, 2629–2640. doi: 10.1093/jxb/eraa059, PMID: 32006044 PMC7210771

[ref81] Wang D. Yeats T. H. Uluisik S. Rose J. K. C. Seymour G. B. (2018). Fruit softening: revisiting the role of pectin. Trends Plant Sci. 23, 302–310. doi: 10.1016/j.tplants.2018.01.006, PMID: 29429585

[ref82] Willats W. G. T. McCartney L. Mackie W. Knox J. P. (2001). Pectin: cell biology and prospects for functional analysis. Plant Mol. Biol. 47, 9–27. doi: 10.1023/a:1010662911148, PMID: 11554482

[ref83] Wolf S. Greiner S. (2012). Growth control by cell wall pectins. Protoplasma 249, 169–175. doi: 10.1007/s00709-011-0371-5 22215232

[ref84] Wolf S. Mouille G. Pelloux J. (2009). Homogalacturonan methyl-esterification and plant development. Mol. Plant 2, 851–860. doi: 10.1093/mp/ssp066, PMID: 19825662

[ref85] Wu H. B. Wang B. Chen Y. Liu Y. G. Chen L. (2013). Characterization and fine mapping of the rice premature senescence mutant ospse1. Theor. Appl. Genet. 126, 1897–1907. doi: 10.1007/s00122-013-2104-y, PMID: 23624440

[ref86] Xiao C. Zhang T. Zheng Y. Cosgrove D. J. Anderson C. T. (2016). Xyloglucan deficiency disrupts microtubule stability and cellulose biosynthesis in Arabidopsis, altering cell growth and morphogenesis. Plant Physiol. 170, 234–249. doi: 10.1104/pp.15.01395, PMID: 26527657 PMC4704587

[ref87] Yang L. Huang W. Xiong F. Xian Z. Su D. Ren M.. (2017). Silencing of SlPL, which encodes a pectate lyase in tomato, confers enhanced fruit firmness, prolonged shelf-life and reduced susceptibility to grey mould. Plant Biotechnol. J. 15, 1544–1555. doi: 10.1111/pbi.12737, PMID: 28371176 PMC5698048

[ref88] Zerzour R. Kroeger J. Geitmann A. (2009). Polar growth in pollen tubes is associated with spatially confined dynamic changes in cell mechanical properties. Dev. Biol. 334, 437–446. doi: 10.1016/j.ydbio.2009.07.044, PMID: 19666018

[ref89] Zhang G. F. Staehelin L. A. (1992). Functional compartmentation of the golgi-apparatus of plant cells: immunocytochemical analysis of high-pressure frozen-substituted and freeze-substituted sycamore maple suspension culture cells. Plant Physiol. 99, 1070–1083. doi: 10.1104/pp.99.3.1070, PMID: 16668973 PMC1080586

[ref90] Zhang T. Tang H. Vavylonis D. Cosgrove D. J. (2019). Disentangling loosening from softening: insights into primary cell wall structure. Plant J. 100, 1101–1117. doi: 10.1111/tpj.14519, PMID: 31469935

[ref91] Zhao F. Chen W. Sechet J. Martin M. Bovio S. Lionnet C.. (2019). Xyloglucans and microtubules synergistically maintain meristem geometry and phyllotaxis. Plant Physiol. 181, 1191–1206. doi: 10.1104/pp.19.00608, PMID: 31537749 PMC6836833

